# Optimization of *In Vitro* Techniques for Distinguishing between Live and Dead Second Stage Juveniles of *Heterodera glycines* and *Meloidogyne incognita*

**DOI:** 10.1371/journal.pone.0154818

**Published:** 2016-05-04

**Authors:** Ni Xiang, Kathy S. Lawrence

**Affiliations:** Department of Entomology and Plant Pathology, Auburn University, Auburn, Alabama, United States of America; East Carolina University, UNITED STATES

## Abstract

*Heterodera glycines* (Soybean Cyst nematode, or SCN) and *Meloidogyne incognita* (Root-Knot nematode, or RKN) are two damaging plant-parasitic nematodes on important field crops. Developing a quick method to distinguish between live and dead SCN and RKN second stage juveniles (J2) is vital for high throughput screening of pesticides or biological compounds against SCN and RKN. The *in vitro* assays were conducted in 96-well plates to determine the optimum chemical stimulus to distinguish between live and dead SCN and RKN J2. Sodium carbonate (Na_2_CO_3_), sodium bicarbonate (NaHCO_3_), and sodium hydroxide (NaOH) were evaluated for the nematode response to see if these compounds can help distinguish between viable from the dead J2. Results indicated that live SCN J2 responded equally (*P* ≤ 0.05) to 1 μl Na_2_CO_3_ and 10 μl NaHCO_3_ in 100 μl of water at pH = 10. Live SCN J2 responded by twisting their bodies in a curling shape and increasing rate of movements within 2 minutes of exposure. The twisting activity continued for up to 30 minutes. Live RKN J2 responded by increasing activity with the application of 1 μl NaOH in 100 μl of water at pH = 10 also in the 2 minutes to 30 minutes time frame. Furthermore, in growth chamber tests to confirm the infectivity of live SCN. The live SCN as determined by exposure to 1 μl of Na_2_CO_3_ indicated 60.5% of the SCN J2 were alive and of those, 29.5% were infective and entered the soybean roots. The 1 μl of NaOH stimulus revealed that 75.2% RKN J2 were alive and of those, 14.9% were infective and entered soybean roots. These results confirmed that 1 μl of Na_2_CO_3_ added to 100 μl suspension of SCN J2 and 1 μl of NaOH added to 100 μl suspension of RKN J2 are the effective stimuli for rapidly distinguishing between live and dead SCN and RKN J2 *in vitro*. SCN and RKN J2 responded differently to different compounds.

## Introduction

Soybean Cyst nematode (SCN), *Heterodera glycines* Ichinohe 1952 and Root-Knot nematode (RKN), *Meloidogyne incognita* (Kofoid & White, 1919) Chitwood 1949 are two plant-parasitic nematodes that cause extensive economic damage to soybean and cotton every year in the U.S. Initial screening of new chemical and biological compounds for management of those nematodes begins with *in vitro* screening of large numbers of samples to determine the best candidates for advancing to greenhouse and field trials. However, distinguishing live from dead J2 with *in vitro* screening is a challenge. Multiple methods have been tried to distinguish between live and dead nematodes either eggs or juveniles. Shepherd [1] found that new blue R can stain the body contents of dead *Tylenchida* while live nematodes remain unstained. Chaudhuri *et al*. [2] stained dead free-living nematode with eosin-Y while live nematodes remained unstained. Ogiga and Estey [3] found that meldola blue and nile blue A are superior and more dependable for distinguishing dead from living nematodes on the specimens of *Dorylaimus*, *Helicotylenchus*, *Mononchus*, *Panagrolaimus*, *Pratylenchus*, *Rhabditis*, *Tylenchorhynchus*, and *Xiphinema* species but not *Heterodera* and *Meloidogyne* species. Meyer *et al*. [4] tested seven different stains on the eggs of *H*. *glycines* and found that chrysoidin, eosin-Y, new blue R, and nile blue A were useful in differentiating dead from live eggs while acridine orange, eosin-Y, fluorescein, and fluorescein diacetate differentially stained live and dead eggs when viewed with fluorescence optics. These staining methods mentioned previously are time-consuming and none of them worked on the live juveniles of SCN or RKN. Faske and Starr [5] tested the sensitivity of *M*. *incognita* and *Rotylenchulus reniformis* to abamectin with concentrations of 21.5, 2.15, 0.22, 0.022, and 0 μg of abamectin/ml *in vitro* in BPI (Bureau of Plant Industries) watch dishes. They distinguished live from dead nematodes by touching each nematode with a small probe [5]. This method is slow and not feasible if many samples or chemicals need to be tested. Bird [6] found that an enzymatically induced fluorescence method using fluorescein diacetate (FDA) can successfully assess the viability of nematodes under UV light. Sample preparation was lengthy for multiple samples. Schroeder and MacGuidwin [7] used fluorescein isothiocyanate (FITC) to distinguish live *H*. *glycines* and found that nematodes incubated in FITC remained active with fluorescence even after two weeks at room temperature, however, not all the nematodes acquired fluorescence quickly or had uniform response. Grego *et al*. [8] found that CellTracker Green labeling (CTG) method was able to distinguish live nematodes from dead anoxia-impacted nematodes. However, all these techniques require time with lengthy sample preparation and expensive fluorescence microscopes which will not facilitate screening large numbers of samples.

Many researchers studied the chemoreception and behavior of free living nematodes and plant parasitic nematodes. Those studies provide a new aspect of using chemical stimuli to distinguish live plant parasitic nematodes from dead ones based on their physiological characteristics. Lee and Atkinson [9] reported that nematodes may respond to stimuli or environmental changes through a sense organ or the nervous system. The behavioral responses of a nematode may be undirected movement under particular stimulation (kinesis) or directed movement with respect to the source of the stimulation (taxis) [9]. They also reported that the bacterial feeder *Caenorhabiditis* is attracted to cyclic nucleotides, certain anions and cations such as Na^+^ and also to basic pH, and that the *Caenorhabiditis* is not attracted to acid pH and the response to hydrate carbon dioxide at concentrations normally found in soils is dependent on the buffer beening used [9]. Sambongi *et al*. [10] also proved that *C*. *elegans* is not attracted to an acidic environment (pH lower that ~4.0) formed by organic or inorganic acids which was dependent on multiple amphid chemo-sensory neurons, and inhibited by a mutation of capsaicin in receptor homologue, and by the addition of amiloride and ruthenium red (inhibitors of proton-gated Na^+^ channels and capsaicin receptors, respectively). Riddle and Bird [11] tested the responses of *R*. *reniformis*, *Anguina agrostis* and *M*. *javanica* to chemical attractants and found that *R*. *reniformis* was attracted to salts and the attractiveness was: Cl^−^ > Na^+^ > C_2_H_3_O_2_^−^ > Mg^2+^, NH_4_^+^, SO_4_^2−^, but *M*. *javanica* J2 were not attracted to the salts. Perry [12] indicated that the sensilla amphids are conserved in a wide range of plant parasitic nematodes including J2 and adult males of RKN, SCN, and *Globodera rostochiensis*, and adults of *Pratylenchus* species, and the chemoreception of nematodes species involved with the amphidial secretions were dissimilar and more specialized in different nematodes. These reports indicated that plant parasitic nematodes may not be attracted to a lower pH environment but respond to a higher pH environment, hydrate carbon dioxide at certain concentrations, and some chemical stimuli. The Na^+^ was previously found to be an attractant for nematodes [9, 11]. Chen and Dickson [13] also found that live juveniles of *H*. *glycines* were able to respond to sodium hydroxide (NaOH) and sodium hypochlorite (NaOCl) by changing the body shape to curl and forming a hook-shape within 30 seconds and the curled body shape lasted for more than 10 minutes, this response was used to determine live from dead J2 of *H*. *glycines*. Chen [14] used NaOH to detect live *H*. *glycines* J2 treated with various fungal culture filtrates. Carbon dioxide (CO_2_) also plays a possible role in attraction of plant parasitic nematodes beyond root exudates and electric potential [15]. Dropkin [15] also reported that exposure to high concentrations of CO_2_ stopped movement of *Heterodera* spp. in a few minutes, and the nematodes recovered promptly upon restoration of oxygen after six hours of exposure to high CO_2_. These literatures suggest that pH, Na^+^, and CO_2_ or HCO_3_^-^ or CO_3_^2-^ may play a possible role in plant parasitic nematode response. The study of the response of the plant parasitic nematodes *H*. *glycines* and *M*. *incognita* to the chemicals Na_2_CO_3_, NaHCO_3_, and NaOH at various pHs will give detailed information about the potential roles of these stimuli for rapidly detecting live or dead SCN and RKN *in vitro*.

The goal of this research was to develop a method to rapidly determine live and dead J2 of SCN and RKN *in vitro*. The specific objectives were: i) to determine the optimum pH that can stimulate a physical response of SCN and RKN J2; and ii) to evaluate the optimum chemical stimuli that elicit a physical response using 20 μl of Na_2_CO_3_, NaHCO_3_, and NaOH for SCN and RKN J2; iii) to evaluate the optimum concentration using the optimum chemical stimuli; iv) to confirm the infectivity and viability of J2 after exposure to the optimum stimulus.

## Materials and Methods

### Nematode and Sodium Solution

#### SCN and RKN J2

SCN eggs were obtained from soybean cysts collected from the 60-d-old soybean stock cultures maintained in 500 cm^3^ polystyrene pots in the greenhouse. Soybean roots were washed through nested 850-μm-pore and 250-μm-pore sieves and cysts were collected from the 250-μm-pore sieve [16]. SCN cysts were ground with a pestle and mortar to release eggs. SCN eggs were obtained after gravitational sieving followed by sucrose centrifugation [17] and recovered on nested 75-μm-pore over 25 -μm-pore sieves. RKN eggs were extracted from the 45-d-old corn stock cultures maintained in 500 cm^3^ polystyrene pots in the greenhouse. Corn roots were rinsed free of the soil, immersed in 0.625% NaOCl solution and shaken at 120 rpm on a rotary shaker for 4 minutes [18]. RKN eggs were cleaned by sucrose centrifugation and collected as described above. SCN and RKN eggs were hatched separately in a modified Baermann funnel which was placed on a Slide Warmer (Model 77) (Marshall Scientific, Brentwood, NH) at 28˚C and 31˚C, respectively [19]. Hatching occurred after 4 to 7 days depending on the season. J2 were collected on a 25-μm-pore sieve, placed in 1.5 ml tubes, centrifuged at 10,000 rpm for 1 minute, washed with distilled sterile water and centrifuged again. Two separate 1.5 ml tubes were prepared with live J2. One tube with live J2 suspension was held at room temperature while the second tube was heated at 65˚C for 5 minutes to kill the J2. Both live and dead J2 suspensions were adjusted to 30 to 40 J2 in 100 μl of water and pipetted into the 96-well plates for the study.

#### Sodium solution

Solutions of 1N Na_2_CO_3_, 1N NaHCO_3_, and 1N NaOH (VWR, Suwanee, GA) were prepared individually by dissolving 21.1, 16.8, or 8.0 g of the compounds, respectively in 200 ml of distilled sterile water. The pH values of these sodium solutions were adjusted to 4, 7, and 10, respectively. 1% acetic acid (CH_3_COOH) was used for pH 4.

### Experiment 1: Determine the Optimum pH of NaHCO_3_ for SCN J2 Responses

Since pH may be an important factor that causes responses in live nematode J2, we tested pH values of 4 with 1% CH_3_COOH, and 7 and 10 with 1N NaHCO_3_ on SCN J2. The experiment was established in 96-well plates. Ten μl of either live, dead or a 50/50 mixture of live and dead SCN J2 suspension containing 30 to 40 J2 and 90 μl of distilled sterile water were pipetted in each well. The treatments were 1% CH_3_COOH at pH 4 and NaHCO_3_ at pH 7 or 10. The experiment was arranged in a randomized complete block design (RCBD) with four replications and the trial was repeated twice.

The J2 were observed at 2, 5, 15, and 30 minutes after exposure under a compound microscope (Nikon TS100) to determine the numbers of live and dead SCN J2 and rated with 1–4 scale within 30 minutes. A rating scale was divided as follows based on the movements and body shapes: 1—no movement of the J2; 2—J2 twitched slowly; 3—J2 moved with normal body shape; 4—J2 twitched quickly with curling body shape. Only 2 and 30 minutes data were presented. Percentages of live J2 were calculated as (live numbers of J2 / Total number of J2) × 100. Rating scales were recorded.

### Experiment 2: Select the Optimum Chemical Stimulus for SCN and RKN J2

The *in vitro* test to determine the best stimulus for physical movement of SCN and RKN J2 responses was conducted. Preliminary experiments indicated that 20 μl of chemicals were the maximum concentration for the three sodium solutions on both SCN and RKN J2. The 20 μl of chemicals 1N Na_2_CO_3_, 1N NaHCO_3_, and 1N NaOH at optimum pH selected in experiment 1 were tested in 96-well plates. Distilled sterile water was used as control. Each well received a 10 μl suspension containing 30 to 40 J2 in a total of 100 μl distilled sterile water. The experiments were arranged in a RCBD with four replications and the trial was repeated twice. Percentages of live J2 were calculated as (live numbers of J2 / Total number of J2) × 100. The J2 were rated using 1–4 scale as described above.

### Experiment 3: Select the Optimum Concentration for the Chemical Stimuli

An *in vitro* test to determine the optimum concentration of the optimum chemical stimulus for live and dead SCN and RKN J2 responses was conducted. The concentrations selected were 1 μl and 10 μl of the chemical at the optimum pH selected in experiments 1 and 2. The test was conducted in 96-well plates *in vitro* as descried previously. The experiments were arranged in a RCBD with four replications and the trial was repeated twice. Percentages of live J2 were calculated as (live numbers of J2 / Total number of J2) × 100. The J2 were rated at 1–4 scale and recorded.

### Experiment 4: Confirm Infectivity and Viability after Exposure to Selected Chemical Stimuli

A bioassay was used to confirm the live J2 were truly alive and infective and the dead J2 were immobile and not infective using soybean plants in the growth chamber. The selected sodium stimuli 1 μl 1N Na_2_CO_3_ and 10 μl 1N NaHCO_3_ in a total of 100 μl water at pH = 10 tested on SCN and 1 μl 1N NaOH in 100 μl of water at pH = 10 tested on RKN were confirmed in growth chamber evaluations using 50 ml conical tubes filled with pasteurized soil. Two seeds of ‘Hutcheson’ soybean (susceptible to both SCN and RKN) were planted and thinned to one seedling in each tube. Six-day-old plants at emergence were inoculated with live or dead SCN or RKN J2 prepared and viability status as described previously. The SCN and RKN J2 live and dead treatments were standardized to 1000 J2 / ml and added to the respective tubes. The actual number of live J2 as determined by the sodium stimuli were calculated as (live numbers J2 / total numbers of J2) × 100. Controls were SCN and RKN live and dead J2 not exposed to sodium stimuli but viability determined by direct observations under the microscope. Plants were incubated at 28°C for SCN and 30°C for RKN in the growth chamber with a 12 hour light and dark phase and watered twice daily as needed for 21 days. Soybean roots were removed, weighed, and stained with acid fuchsin at 21 days after inoculation (DAI). Acid fusion stain by Byrd et al. 1983 [20] was used to stain the SCN J2 in the soybean roots to confirm the SCN J2 were alive and had enter the roots to begin feeding. The J2 in the roots were enumerated using a dissection microscope (Nikon SMZ800) at 10X. Percentages of J2 enumerated in the roots were calculated as (numbers of J2 in the roots/number of live J2 at inoculation) ×100. The experiments were arranged in a RCBD with five replications and the trial was repeated twice.

### Data Analysis

Data on *in vitro* tests and soybean root bioassays were analyzed with SAS 9.4 software (SAS Institute, Carry, NC) using Glimmix procedure. Student panel graphs were generated to test the normality of the data. Treatment LS-means were compared by Tukey-Kramer’s method at the significant level of *α* ≤ 0.05. Data from two repeated trials were analyzed separately to determine any interactions over time prior to pooling if there was no interaction.

## Results

### Results of Experiment 1: Optimum pH of NaHCO_3_ for SCN J2 Responses

Live SCN J2 responded differently to the solutions with pH 4 of 1% CH_3_COOH and pH 7 and 10 of 1N NaHCO_3_. The pH = 4 solution increased the movement of live SCN J2 by 5.6% at 2 minutes indicating the nematodes were alive (*P* ≤ 0.05) (Table 1, S1 Table). However, SCN J2 movement decreased significantly (*P* ≤ 0.05) from the 5 to 30 minutes time. The pH = 7 did not stimulate SCN J2 movement at 2 minutes through the 30 minutes rating time periods (Table 1). The pH = 10 solutions stimulated an increase of the SCN J2 movement by 8.5% at 2 minutes which was similar to that observed by pH = 4. However, pH = 10 continued to stimulate SCN J2 movement through the 30 minutes observation period with a significant higher rating scale (*P* ≤ 0.05) (Table 1). Dead J2 didn’t respond to any pH test solutions and the J2 remained motionless (Table 1). These results indicated that pH = 10 is the optimum pH value to evaluate SCN J2 response for determining that SCN J2 were alive and moving or dead and immobile. The pH = 10 would be used in the following trials.

**Table 1:** Percentages of live SCN J2 under different pH values over time in 30 minutes.

**pH value**	SCN J2	Before exposure	2 mins exposure	30 mins exposure	% live J2 changed at 2 mins	Rating at 2 mins	% live J2 changed at 30 mins	Rating at 30 mins
		% live J2	% live J2	% live J2				
**pH = 4 1% CH**_**3**_**COOH**	Live	84.0	89.6	9.9	5.6	3.9 a*	-74.1 c*	1.2 d*
	Live/Dead	42.4	47.4	16.2	5.0	3.7 ab	-26.2 b	1.7 c
	Dead	0.0	0.0	0.0	0.0	1.1 d	0.0 a	1.1 d
**pH = 7 1N NaHCO**_**3**_	Live	86.2	85.1	58.8	-1.1	2.8 c	-27.4 b	2.0 c
	Live/Dead	46.6	46.7	46.7	0.1	2.8 c	0.1 a	2.8 b
	Dead	0.0	0.0	0.0	0.0	1.1 d	0.0 a	1.1 d
**pH = 10 1N NaHCO**_**3**_	Live	83.7	92.2	92.2	8.5	3.4 b	8.5 a	3.9 a
	Live/Dead	41.1	46.3	46.9	5.2	3.8 a	5.8 a	3.9 a
	Dead	0.0	0.0	0.0	0.0	1.1 d	0.0 a	1.1 d

*LS-MEANS with the same letter are not significantly different according to Tukey-Kramer’s method (*P* ≤ 0.05). There was no treatment x trial interactions thus data was combined for analysis. Data presented are average of two trials with a total of 8 replications for each treatment.

### Results of Experiment 2: Select the Optimum Chemical Stimulus for SCN and RKN J2

#### SCN J2

**Fig 1:**
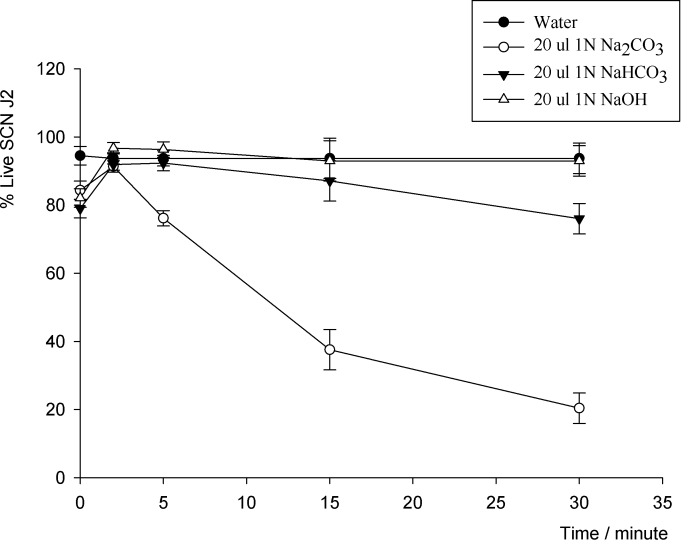
SCN J2 responded to three sodium chemicals, 1N Na_2_CO_3_, 1N NaHCO_3_, and 1N NaOH at 20 μl application.

The three chemicals 1N Na_2_CO_3_, 1N NaHCO_3_, and 1N NaOH at pH = 10 tested at 20 μl caused different responses on live SCN J2 at different time points which visibly distinguished live from dead J2. The three chemicals were equally effective (*P* ≤ 0.05) at distinguishing live from dead SCN J2 from the 0 to 2 minutes time period. With longer exposure time, 20 μl Na_2_CO_3_ appeared toxic to the SCN J2 and a significant decrease (*P* ≤ 0.05) in movement of the nematodes was observed at 30 minutes (Fig 1, S1 Fig). The 20 μl NaHCO_3_ slightly decreased the movement of the nematode with increase time (Fig 1).

### RKN J2

**Fig 2:**
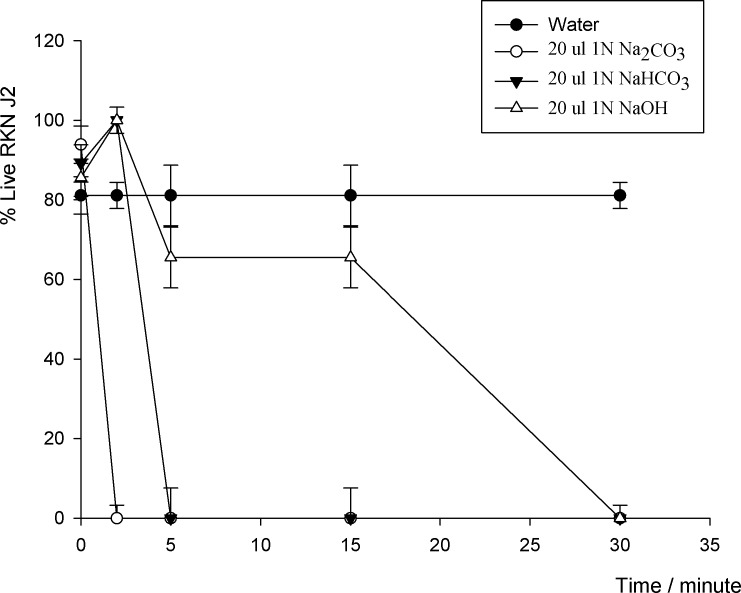
RKN J2 responded to three sodium chemicals, 1N Na_2_CO_3_, 1N NaHCO_3_, and 1N NaOH at 20 μl application.

The 1N Na_2_CO_3_ was highly toxic to RKN J2 which caused a significant decrease in movement of RKN J2 (*P* ≤ 0.05) within 2 minutes exposure to the chemical (Fig 2, S2 Fig). The NaHCO_3_ and NaOH stimulated the movements of RKN J2 at 2 minutes (Fig 2) with distinctive curling or hooked body shapes. However, the NaHCO_3_ caused significant decreasing movement of RKN J2 after 2 minutes exposure (*P* ≤ 0.05). The NaOH also slightly decreased the movement of RKN J2 from 2 minutes to 15 minutes period of time.

### Results of Experiment 3: Select the Optimum Concentration for the Chemical Stimuli

#### SCN J2

**Fig 3:**
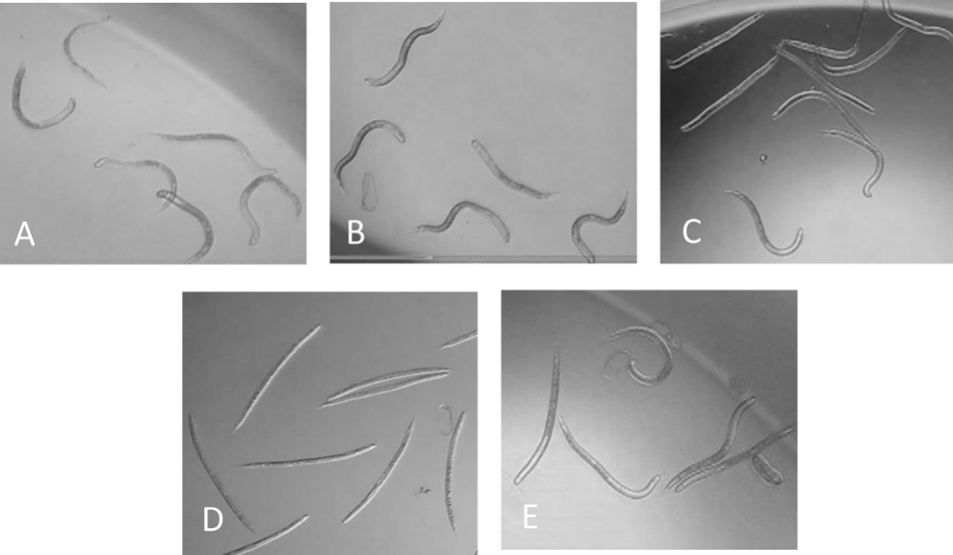
Responses of SCN J2 to test agents at 30 minutes. SCN J2 response to 1 μl 1N Na_2_CO_3_ at 30 minutes (A); SCN J2 response to 10 μl 1N NaHCO_3_ at 30minutes (B); SCN J2 in water control at 30 minutes (C); Dead SCN J2 did not response to any test agent (D); SCN J2 response to 10 μl 1N NaOH at 30 minutes (E).

In previous experiments, the 20 μl dose of treatments was toxic and a lower volume of the test solutions was evaluated. The optimum concentration for SCN J2 tested was from 1 μl and 10 μl of 1N Na_2_CO_3_, 1N NaHCO_3_, and 1N NaOH at pH = 10. The 1 μl of Na_2_CO_3_, NaHCO_3_, and NaOH stimulated movement of SCN J2 within 2 minutes exposure, but only 1 μl of Na_2_CO_3_ and 10 μl NaHCO_3_ caused live SCN J2 to rapidly curl and twist into a hooked shape (Fig 3A and 3B) after 2 minutes exposure which was easily distinguished live from dead J2 (Table 2, S2 Table, Fig 3C and 3D). However, the 10 μl volume caused J2 to float and therefore compounded counting. The NaOH did not cause the live SCN J2 to curl and twist (Fig 3E). The 1 μl of Na_2_CO_3_ was optimum for distinguishing between live and dead SCN J2 in 30 minutes and was tested in the growth chamber.

**Table 2:** Response of SCN J2 to 1 or 10 μl of 1N Na_2_CO_3_, 1N NaHCO_3_, and 1N NaOH solutions at pH = 10.

Sodium stimuli	Volume/μl	Before exposure	2 mins exposure	30 mins exposure	% changed live SCN J2 at 2 mins	Rating scale at 2 mins	% changed live SCN J2 at 30 mins	Rating scale at 30 mins
		% live J2	% live J2	% live J2				
**1N Na**_**2**_**CO**_**3**_	1	87.7	92.4	93.6	4.7 abc*	3.8 a*	5.9 ab*	3.8 a*
	10	81.0	91.4	46.4	10.4 ab	3.5 ab	-34.6 b	1.2 d
**1N NaHCO**_**3**_	1	80.5	81.6	79.8	1.1 bc	3.0 bc	-0.7 ab	1.9 c
	10	86.2	93.5	96.8	7.3 abc	3.5 ab	10.6 ab	3.3 ab
**1N NaOH**	1	79.2	85.9	71.7	6.7 abc	3.3 abc	-7.5 b	2.1 c
	10	78.6	92.9	91.7	14.3 a	3.6 a	13.1 a	2.8 b
**Water Control**	100	94.5	93.7	93.7	-0.8 c	2.9 c	-0.8 ab	2.9 b

*LS-MEANS with the same letter are not significantly different according to Tukey-Kramer’s method (*P* ≤ 0.05). There was no treatment x trial interactions thus data was combined for analysis. Data presented are average of two trials with a total of 8 replications for each treatment.

#### RKN J2

**Fig 4:**
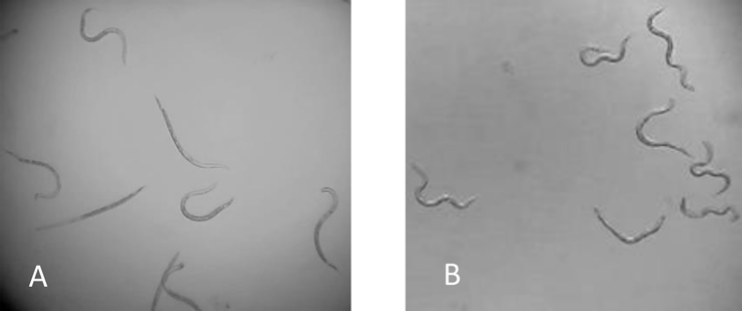
Responses of RKN J2 to water and 1 μl of 1N NaOH at 30 minutes. RKN J2 in water with normal movement at 30 minutes (A); RKN J2 exposed in 1 μl of 1N NaOH with curled and twisted body shape at 30 minutes(B).

The optimum concentration for RKN J2 was selected from 1 μl and 10 μl of 1N NaOH at pH = 10. The 1μl NaOH caused significant increasing movement of RKN J2 at 30 minutes (*P* ≤ 0.05) with distinctive curled and twisted body shapes (Table 3, S3 Table, Fig 4A and 4B). The 10 μl of NaOH was toxic to the RKN J2 at 30 minutes and the 10 μl volume caused floating which is not advantageous for *in vitro* screening (Table 3). The 1 μl of NaOH was chosen and tested in the growth chamber.

**Table 3:** Response of RKN J2 to different concentrations of 1N NaOH at pH = 10.

Sodium stimuli	Volume/μl	Before exposure	2 mins exposure	30 mins exposure	% changed live RKN J2 at 2 mins	Rating scale at 2 mins	%changed live RKN J2 at 30 mins	Rating scale at 30 mins
		% live J2	% live J2	% live J2				
**1N NaOH**	1	77.7	99.5	99.5	21.8	3.8 a	21.8 a*	3.8 a
	10	77.0	100.0	67.6	23.0	2.7 b	-9.4 c	1.3 c
**Water Control**		81.1	81.1	81.1	0.0	2.9 ab	0.0 bc	2.9 b

*LS-MEANS with the same letter are not significantly different according to Tukey-Kramer’s method (*P* ≤ 0.05). There was no treatment x trial interactions thus data was combined for analysis. Data presented are average of two trials with a total of 8 replications for each treatment.

### Results of Experiment 4: Confirm Infectivity and Viability after Exposure to Selected Chemical Stimuli

#### SCN in soybean

**Fig 5:**
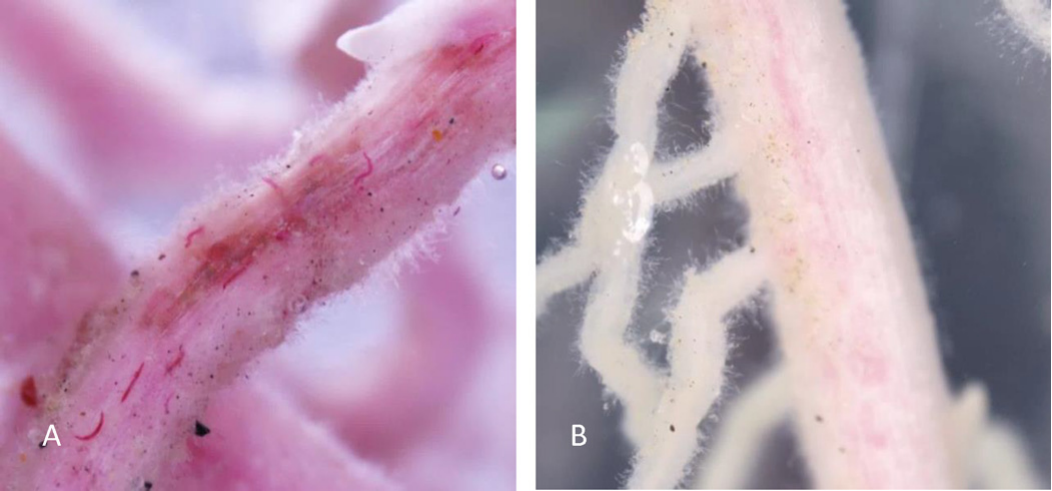
Stained SCN J2 in the root tissues from a live SCN treatment at 21 DAI (A) and the stained roots from the dead SCN treatments with no SCN J2 or females (B).

Results indicated that the 1μl 1N Na_2_CO_3_ in 100 μl of solution at pH = 10 indicated that 60.5% of the SCN J2 were alive and of these 29.5% SCN J2 entered the soybean roots (Table 4, Fig 5A). All of the SCN J2 which were determined to be dead by the sodium solution when added to plants were not infective as indicated by their inability to enter the roots and none were observed within the root tissue (Table 4, Fig 5B). Soybean root fresh weights were similar among all the treatments (Table 4). The 1μl 1N Na_2_CO_3_ was the best indicator of live SCN J2 and J2 were infective entering soybean roots.

**Table 4:** SCN J2 infection of soybean roots after exposed to 1 μl 1N Na_2_CO_3_ live and dead determination.

SCN J2	Sodium stimuli	Volume/μl	Percent live J2 inoculated	Percent Females and J2 in roots at 21 DAI	Root fresh weight at 21 DAI/g
**Live**	1N Na_2_CO_3_	1	60.5 a	29.5 a	1.5 a*
	Water Control		57.6 a	17.0 ab	1.4 a
**Dead**	1N Na_2_CO_3_	1	1.1 b	0.0 b	1.9 a
	Water Control		0.0 b	0.0 b	1.7 a

*LS-MEANS with the same letter are not significantly different according to Tukey-Kramer’s method (*P* ≤ 0.05). There was no treatment x trial interactions thus data was combined for analysis. Data presented are average of two trials with a total of 10 replications for each treatment. Percentage of live SCN J2 inoculated with soybean roots and SCN J2 penetrated in the roots at 21 DAI.

#### RKN in soybean

**Fig 6:**
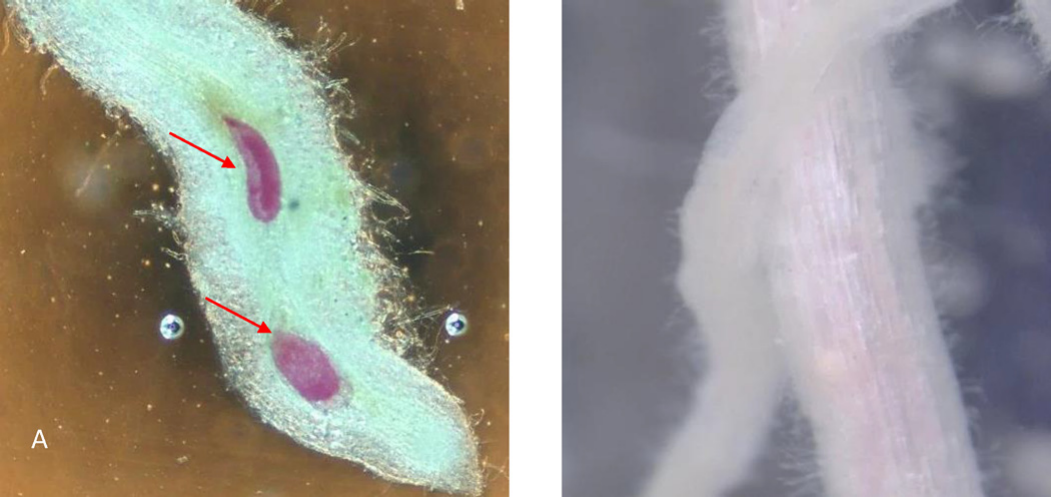
Stained RKN female with eggs in the root tissues from a live RKN treatment at 21 DAI (A) and the stained root tissues from the dead RKN treatments with no RKN J2 or females (B).

Growth chamber results of the 1μl of 1N NaOH in 100 μl of solution at pH = 10 indicated 75.2% of RKN J2 were alive and 14.9% J2 entered the roots (Table 5, Fig 6A). RKN J2 and females were recorded at 21DAI. Dead RKN J2 were confirmed dead and were not infective as measured by their absence in the roots (Table 5, Fig 6B). The root fresh weights were similar among all the treatments (Table 5). Thus, 1 μl 1N NaOH at pH = 10 is the best indicator for live RKN J2 and J2 were infective in soybean roots.

**Table 5:** RKN J2 infection of soybean roots after 1 μl 1N NaOH live and dead determination.

RKN J2	Sodium stimuli	Volume/μl	Percent live J2 inoculated	Percent females and J2 in roots at 21 DAI	Root fresh weight at 21 DAI / g
**Live**	1N NaOH	1	75.2 a	14.9 a	1.4 a*
	Water control		66.5 a	8.0 ab	1.3 a
**Dead**	1N NaOH	1	1.7 b	0.0 b	1.4 a
	Water Control		1.9 b	0.0 b	1.5 a

*LS-MEANS with the same letter are not significantly different according to Tukey-Kramer’s method (*P* ≤ 0.05). There was no treatment x trial interactions thus data was combined for analysis. Data presented are average of two trials with a total of 10 replications for each treatment. Percentage of live RKN J2 inoculated in the roots and RKN females and J2s penetrated in the roots at 21 DAI.

## Discussion

The pH test indicated that live SCN J2 responded to the higher pH rather than the lower ones. The pH = 10 can successfully distinguish between live and dead SCN J2 in 30 minutes *in vitro*. Chen and Dickson [13], previously reported pH about 12.3 effectively stimulated SCN J2 but theorized the response was because of toxic action of NaOH. Sambongi *et al*. [10] also proved that *C*. *elegans* is not attracted to an acidic environment with pH lower than 4.0 formed by organic or inorganic acids. Our experiments demonstrated that high pH = 10 effectively stimulated SCN J2, but low pH did not cause any response on SCN J2. The same response has also been found on RKN J2 (data not shown). We proved that SCN and RKN physically respond to a high pH. The pH value plays an important role in stimulating nematode responses.

Results of selecting the optimum stimuli *in vitro* and in growth chamber bioassay revealed that SCN J2 responded to 1 μl 1N Na_2_CO_3_ and RKN J2 responded to 1 μl 1N NaOH in 100 μl of water at pH = 10 indicated 1 μl 1N Na_2_CO_3_ and 1 μl 1N NaOH in 100 μl of water at high pH are the best indicators to distinguishing between live and dead SCN and RKN J2 *in vitro*, respectively. Nehrke and Melvin [21] found that the NHX-4, one of the nine putative homologs of *C*. *elegans* and the ubiquitous nematode Na^+^-H^+^ exchanger, mediates Na^+^-dependent pH recovery after intracellular acidification. In our study, adding Na^+^ and raising the pH around the nematodes may contribute to the stimulation of SCN and RKN J2 through the Na^+^-H^+^ exchanger in the nematodes but more research is needed to prove that. Perry [12] mentioned the role and functioning of the anterior chemosensory organs of plant parasitic nematode especially the amphids and found that the amphidial secretions were involved in the chemoreception and the behavioral of nematode responses to semiochemicals such as sex pheromones. In addition, amphids, which are the largest and most complex of the anterior sensilla, are conserved in many plant parasitic nematodes including J2 and adult males of *M*. *incognita*, *H*. *glycines*, and other nematodes [12, 22–23]. This information indicated that the responses of SCN and RKN J2 to the high pH and Na_2_CO_3_ or NaOH are possibly involved with SCN and RKN J2 chemosensory organs and with the amphidial secretions which play an important role in chemoreception [12].

Overall, this sodium technique is very accurate at determining live and dead nematodes when applied *in vitro* to test the efficacy of nematicides or biocontrol agents and can be used for high throughput screening. The application of stimuli is a very easy and simple screening method not requiring the special training for sample preparation nor advanced equipment [6–8]. The quick consistent responses of the live nematodes to the sodium stimuli consistently indicates efficacy of the tested agents. The other techniques [6–8] of dyes or labeling materials cannot guarantee all the nematode will be labelled the same in a short time period. Health and safety concerns are also present with the fluorescent materials FDA, FTIC, and CTG as well as availability of fluorescence microscopes. The application of 1 μl Na_2_CO_3_ or NaOH can not only distinguish between live and dead nematodes, but also are relatively safe to use. Beyond SCN and RKN J2, Lesion nematode J2 and adults also responded to the 1 μl Na_2_CO_3_ at pH = 10 (data not presented).

## Conclusions

Results from this research clearly demonstrate that applying 1 μl 1N Na_2_CO_3_ in 100 μl SCN solution at pH = 10 and 1 μl 1N NaOH in 100 μl RKN solution at pH = 10 can be quick and useful methods for high throughput screening chemical or biological agents of SCN or RKN *in vitro*. It is an easy, practical, and economical method. Using this method we screened 700 bacterial strains for efficacy to SCN and RK in 3 months.

## Supporting Information

S1 Fig Original data set for Fig 1. (XLSX)

S2 Fig Original data set for Fig 2. (XLSX)

S1 Table Original data set for Table 1. (XLSX)

S2 Table Original data set for Table 2. (XLSX)

S3 Table Original data set for Table 3. (XLSX)
